# 1240. Ceftobiprole Activity against Drug-Resistant *Staphylococcus aureus* Clinical Isolates Collected in the United States from 2016 through 2020

**DOI:** 10.1093/ofid/ofab466.1432

**Published:** 2021-12-04

**Authors:** Leonard R Duncan, Kamal Hamed, Jennifer Smart, Michael A Pfaller, Helio S Sader

**Affiliations:** 1 JMI Laboratories, North Liberty, Iowa; 2 Basilea Pharmaceutica International Ltd., Basel, Basel-Stadt, Switzerland; 3 Basilea Pharmaceutica International AG, Basel, Basel-Stadt, Switzerland

## Abstract

**Background:**

Multidrug-resistant (MDR) and methicillin-resistant *Staphylococcus aureus* (MRSA) present significant treatment challenges and can cause serious morbidity and mortality. Ceftobiprole, the active moiety of the prodrug ceftobiprole medocaril, is an advanced cephalosporin approved in many European and other countries for the treatment of adults with community- and hospital-acquired pneumonia, excluding ventilator-associated pneumonia. Ceftobiprole is currently in phase 3 clinical development to support a New Drug Application in the United States for acute bacterial skin and skin structure infections and *S. aureus* bacteremia. Here, the activity of ceftobiprole and comparators was evaluated against recent MDR *S. aureus* and MRSA clinical isolates.

**Methods:**

13,868 *S. aureus* isolates were collected from patients with various infection types at 34 US medical centers from 2016–2020. Susceptibility to ceftobiprole and comparator agents was tested by CLSI methods. Current CLSI and EUCAST interpretive criteria were applied (Table). Isolates were categorized as MDR if they were non-susceptible (NS; CLSI criteria) to ≥3 of the following antimicrobials: clindamycin (CM), daptomycin (DAP), erythromycin (ERY), gentamicin (GM), levofloxacin (LEV), linezolid (LZD), tetracycline (TET), tigecycline (TGC), trimethoprim-sulfamethoxazole (TMP-SMX), or vancomycin (VAN). Isolates displaying oxacillin MIC values ≥4 mg/L were categorized as MRSA.

**Results:**

Ceftobiprole was more active than ceftaroline (CPT) against MRSA (99.2% susceptible [S] versus 94.0% S, respectively) (Table). Ceftobiprole maintained activity against 88.0% of the CPT-NS isolates, but CPT was only active against 6.5% of the ceftobiprole-NS isolates. Ceftobiprole was also highly active (97.7–100.0% S) against isolates NS to CM, DAP, ERY, GM, LEV, LZD, TET, TGC, or TMP-SMX. No VAN-NS isolates were detected. Importantly, ceftobiprole was more active (97.7% S) than CPT (83.0% S) against the subset of MDR-MRSA isolates.

**Conclusion:**

**Conclusions**: Ceftobiprole was highly active *in vitro* against MRSA and MDR *S. aureus* collected at US medical centers during 2016–2020. These results support the further development of ceftobiprole to treat *S. aureus* infections in the US.

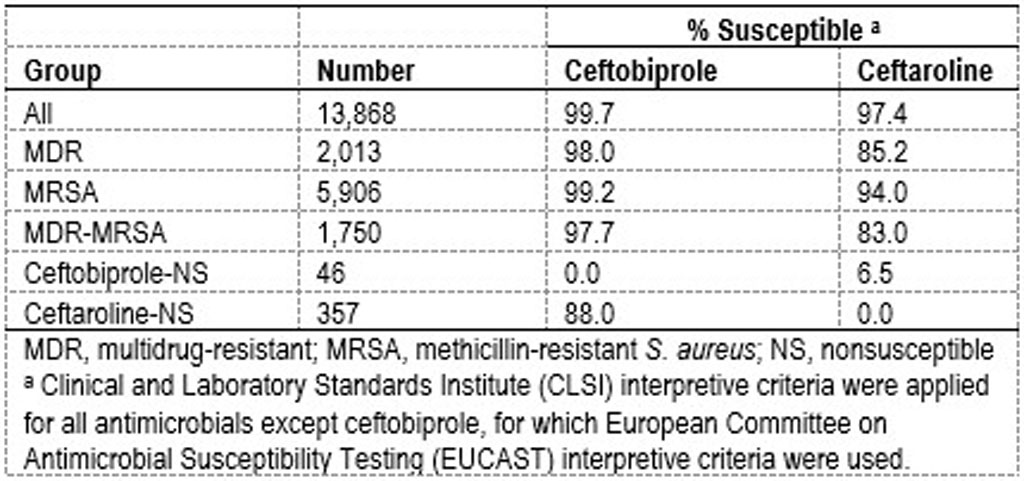

**Disclosures:**

**Leonard R. Duncan, PhD**, **AbbVie (formerly Allergan**) (Research Grant or Support)**Basilea Pharmaceutica International, Ltd.** (Research Grant or Support)**Cipla Therapeutics** (Research Grant or Support)**Cipla USA Inc.** (Research Grant or Support)**Department of Health and Human Services** (Research Grant or Support, Contract no. HHSO100201600002C)**Shionogi** (Research Grant or Support) **Kamal Hamed, MD, MPH**, **Basilea Pharmaceutica International, Ltd** (Employee)**Department of Health and Human Services** (Research Grant or Support, Contract no. HHSO100201600002C) **Jennifer Smart, PhD**, **Basilea Pharmaceutica International, Ltd.** (Employee)**Department of Health and Human Services** (Research Grant or Support, Contract no. HHSO100201600002C) **Michael A Pfaller, MD**, **Basilea Pharmaceutica International, Ltd.** (Research Grant or Support)**Cidara Therapeutics, Inc.** (Research Grant or Support)**Department of Health and Human Services** (Research Grant or Support, Contract no. HHSO100201600002C)**Pfizer, Inc.** (Research Grant or Support) **Helio S. Sader, MD, PhD, FIDSA**, **AbbVie (formerly Allergan**) (Research Grant or Support)**Basilea Pharmaceutica International, Ltd.** (Research Grant or Support)**Cipla Therapeutics** (Research Grant or Support)**Cipla USA Inc.** (Research Grant or Support)**Department of Health and Human Services** (Research Grant or Support, Contract no. HHSO100201600002C)**Melinta Therapeutics, LLC** (Research Grant or Support)**Nabriva Therapeutics** (Research Grant or Support)**Pfizer, Inc.** (Research Grant or Support)**Shionogi** (Research Grant or Support)**Spero Therapeutics** (Research Grant or Support)

